# Sense of agency modulates contextual learning in visual search

**DOI:** 10.1007/s00426-026-02368-5

**Published:** 2026-08-29

**Authors:** Siyi Chen, Nele Anna Wunner, Puyuan Liu, Simone Schütz-Bosbach

**Affiliations:** Neuro-Cognitive Psychology, Department of Psychology, LMU München, Leopoldstr. 13, D-80802 Munich, Germany

## Abstract

Sense of Agency (SoA) refers to the subjective experience of control over one’s actions and their outcomes. Although agency-related action–outcome processes have been shown to influence attention, memory, and learning, it remains unclear whether the reliability of action-linked perceptual consequences shapes contextual learning. The present study examined whether contextual cueing—a form of learning in which repeated spatial layouts facilitate visual search—is modulated by two aspects of the action–outcome context. Experiment 1 compared a Free-Control condition, in which responses immediately terminated the search display, with a Constrained-Control condition, in which display offset was externally determined. Contextual cueing developed more strongly when responses reliably produced display offset. Experiment 2 tested whether configuration-specific contingencies between responses and subsequent visual outcomes modulate contextual learning. Contextual cueing was larger when old displays were more reliably followed by a response-triggered visual outcome, even though the overall frequency of such outcomes was held constant across sessions. Together, the findings provide preliminary evidence that contextual learning is sensitive to the reliability of action-linked perceptual consequences. Reliable response–effect relations and configuration-specific outcome contingencies may support the updating and stabilization of context–target associations across repeated encounters.

**Supplementary Information:**

The online version contains supplementary material available at https://doi.org/10.1007/s00426-026-02368-5.

## Introduction

Contextual cueing is a paradigm used to study spatially statistical learning. In contextual cueing tasks, participants search for a target among distractors, and the repeated spatial arrangement of distractors facilitates faster target detection over time, reflecting implicit learning of the spatial configuration — the contextual cueing effect (Chun & Jiang, 1998). The contextual cueing effect is often described as automatic; recent studies with fewer repeated patterns show that participants can sometimes explicitly recognize familiar configurations (Chen et al. 2025a, b). Within a hierarchical predictive-coding perspective (Friston, 2010), contextual cueing reflects the influence of long-term memory representations that generate predictions about likely target and distractor locations. These top-down expectations are propagated through the visual hierarchy, such that matches between predicted and observed spatial layouts reduce prediction error and strengthen the underlying memory traces. When displays deviate from expectations, increased prediction error drives updating of the internal model and adjusts attentional allocation. From this view, contextual cueing can shape processing at early perceptual stages, guiding attention toward predicted locations (Chun & Jiang, 1998; Chaumon et al., 2008; Chen et al., 2024; Johnson et al., 2007; Zinchenko et al., 2020). Alternative accounts propose that contextual cueing may also operate at later stages, facilitating response mapping or motor selection once the target has been identified (Chen et al., 2021; Hout & Goldinger, 2012; Kunar et al., 2007). However, it remains unclear whether contextual cueing is systematically shaped by action–outcome processes that contribute to the sense of agency (SoA).

This question bridges contextual-learning research with work on SoA and action–outcome learning. Contextual-cueing accounts typically emphasize how repeated spatial layouts guide attention and facilitate search, but they rarely consider whether the action–outcome context in which these layouts are encountered influences the acquisition of context–target associations. Conversely, SoA theories emphasize that reliable action effects, expected controllability, and outcome monitoring can shape attention, memory, and learning (Huffman and Brockmole 2020; Hon and Yeo 2021; Ren et al. 2023a, b; Wen & Haggard, 2018; Zou et al., 2023), but these ideas have rarely been tested in contextual cueing, where context learning, attentional guidance, and memory-based facilitation are closely intertwined.

The sense of agency (SoA) refers to the subjective experience of controlling one’s own actions and influencing external events (Haggard, 2017). A strong SoA arises when predicted and actual outcomes of one’s actions align, minimizing prediction error and signaling that internal forward models are accurate (Frith et al., 2000; Sato & Yasuda, 2005). Specifically, agency-related signals may influence contextual learning across repeated search episodes. First, the experienced reliability of action effects can establish a state of expected controllability, which may modulate attentional engagement during subsequent search trials (Haggard, 2017; Synofzik et al., 2008; Wen & Haggard, 2018). Second, prediction errors associated with response outcomes may contribute to post-response updating or consolidation of the just-processed configuration, thereby influencing how strongly context–target associations are stored and expressed on later repetitions (Friston, 2010; Hommel, 2009; Waszak et al., 2012). Thus, SoA may shape contextual cueing by modulating the accumulation and stabilization of contextual associations over time, consistent with accounts of contextual cueing as gradual learning across repeated encounters (Chun & Jiang, 1998; Geyer et al., 2010; Sisk et al., 2019).

In this study, we investigated whether and how agency-related manipulations influence contextual learning in a visual-search task. On each trial, participants searched for a rotated T target among L-shaped distractors and indicated the target orientation by pressing one of two response keys. Repeated spatial configurations could facilitate target detection over time, producing contextual cueing. We focused on two complementary dimensions consistent with hierarchical agency models (Haggard, 2017; Synofzik et al., 2008). The first dimension, action-effect control, refers to the reliability and temporal predictability with which one’s actions produce perceptual consequences. In the present task, this was operationalized as whether the response keypress that indicated the target orientation also directly determined when the search display disappeared. This manipulation targets the coupling between an action and its immediate sensory outcome, but it may also influence related aspects of action–outcome processing, including trial fluency, expected controllability, and post-response engagement. In this sense, action-effect control is not restricted to the subjective feeling of control alone, but reflects a broader agency-related control context in which actions either do or do not produce reliable perceptual consequences. The second dimension, outcome-based agency cues, concerns the reliability with which a correct response is followed by performance outcome for particular display types. In this way, it provides configuration-specific evidence about action–outcome contingencies. Together, these two dimensions target agency-related components of action–outcome processing that may modulate the acquisition and expression of contextual learning.

Experiment 1 examined whether contextual learning depends on whether participants’ responses reliably produce an immediate perceptual consequence. We contrasted a Free-Control condition, in which a keypress immediately terminated the search display, with a Constrained-Control condition, in which display offset was determined externally and was therefore not contingent on the participant’s response. Motor responses were possible in both conditions, but only in the Free-Control condition did the response reliably produce display offset. In the Test phase, all trials were performed under Free-Control conditions. This design allowed us to assess whether differences established during training persisted after response-dependent display offset was restored. If reliable action-linked perceptual consequences support the acquisition of contextual associations, contextual cueing should develop more strongly during Free-Control training and this difference may persist into the Test phase. If the manipulation mainly affects the immediate expression or execution of learned responses, the condition difference should diminish once Free Control is restored.

Experiment 2 investigated whether configuration-specific outcome contingencies act as agency-related cues that modulate contextual learning. The likelihood of receiving feedback after responses was varied trial-by-trial for old versus new displays in different sessions. At the same time, we kept the overall frequency of feedback constant across sessions. Specifically, in one session, old configurations were more likely than new configurations to be followed by feedback (75% vs. 25%), whereas in the other session this contingency was reversed (25% vs. 75%). From a predictive-coding view, asymmetric contingencies (75% vs. 25% for old vs. new) should lead the system to treat old displays as more tightly linked to action outcomes, strengthening contextual learning. Accordingly, we expected contextual cueing to be greater when old configurations were more reliably followed by feedback.

Together, these two experiments target agency-related components of action–outcome processing that may influence contextual learning: Experiment 1 tested whether a response reliably produced an immediate perceptual consequence, whereas Experiment 2 tested whether particular contexts were reliably followed by a response-triggered visual outcome.

## Experiment 1

### Methods

#### Participants

Twenty-four right-handed volunteers with normal or corrected-to-normal vision participated in the experiment (10 men, *M* = 22.3 years). We determined the sample size based on a meta-analysis of the standard contextual cueing effect (*d*_*z*_ = 0.97; Vadillo et al., 2021). A power analysis for a two-tailed paired-sample t-test (α = 0.05, power = 0.90, *d*_*z*_ = 0.97) indicated that a minimum of 14 participants was required to detect the effect. The study was approved by the Ethics Committee of the Department of Psychology, LMU Munich. All participants provided written informed consent prior to the experiment and received 9 Euros per hour for their service.

#### Apparatus

The experiment took place in a dark cabin (ambient lighting: 0.35 cd/m^2^). Participants viewed the display screen from a distance of 55 cm. Stimuli were presented on a 19-inch CRT color monitor with a resolution of 1024 × 768 pixels at a refresh rate of 85 Hz. Event scheduling and response recording were controlled by customized Matlab code, using the Psychophysics Toolbox (Psychtoolbox-3.0.18; Brainard, 1997; Pelli, 1997).

#### Stimuli

Each search display—a single visual array presented on the screen during a trial—consisted of 16 gray items (25.38 cd/m^2^; each subtending 1.0° of visual angle) presented against a dark background (1.76 cd/m^2^): 15 of them being L-shaped distractors, randomly rotated in four possible directions (0°, 90°, 180°, or 270°, respectively), and one T-shaped target, rotated either 90° or 270° (i.e., pointing to the left or right). The items were randomly placed at 64 possible locations around four concentric (invisible) circles (with radii of 2°, 4°, 6°, and 8° of visual angle, respectively), though each display quadrant contained the same number of distractor items to avoid salient clusters. To minimize the influence of target eccentricity on search performance, the target item could appear only on the second or the third ring (i.e., 32 possible locations in principle).

#### Design and procedure

The experiment was divided into a training phase and a test phase, each consisting of 240 trials. The training phase comprised two types of blocks: one containing both old and new displays under a free-control condition, and another containing both old and new displays under a constrained-control condition. These blocks were randomly interleaved throughout the experiment.

In old displays, the spatial configuration of all items remained constant across repetitions. In new displays, all distractors appeared at random locations across trials. The target appeared at a fixed, yet distinct, position in each quadrant for both old and new displays. This ensured that performance gains reflected learning of spatial context–target associations, rather than repeated target locations (Geng & Behrmann, 2005).

Four old displays were assigned to the free-control condition (high sense of control), and the other four to the constrained-control condition (low sense of control). To balance action control effects across target locations, four of the target locations in the new displays were assigned to the free-control condition, and the other four to the constrained-control condition. Finally, the assignment of repeated displays and target locations was counterbalanced across participants.

**Fig. 1:**
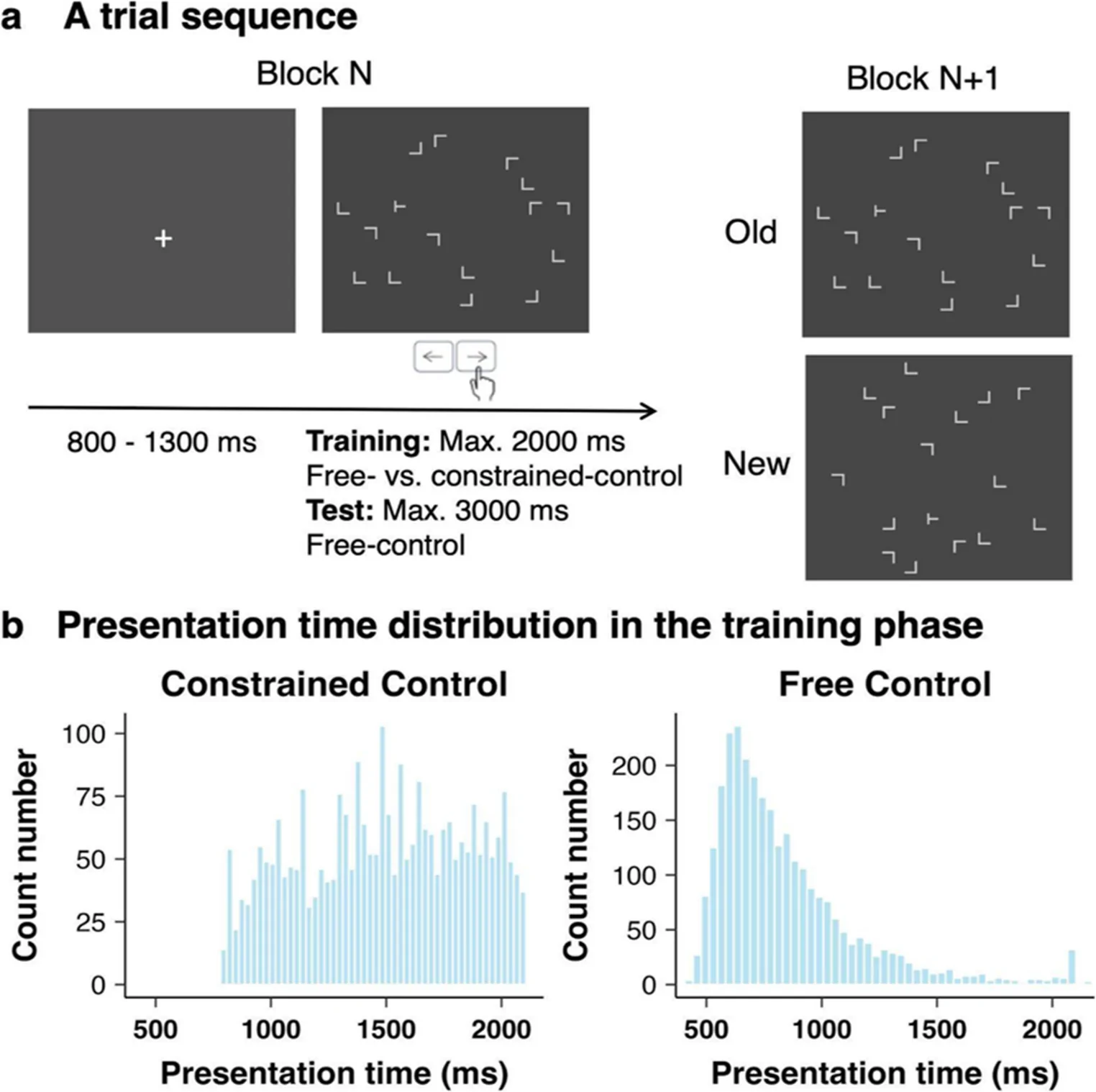
**a** Event sequence on a trial in the experimental paradigm. In free-control trials of training, the display terminated upon response or timeout after 2s. In constrained-control trials of training, the display appeared randomly for 800 ms to 2 s and remained on-screen regardless of response. In the test phase, all trials terminated automatically either after 3 s or immediately upon the response made within the 3-s period. **b** Distribution of presentation times during the training phase for each control condition. The x-axis represents individual trial presentation times, and the y-axis indicates the number of trials (count) per bin. Each histogram uses 50 bins with light blue bars

Participants were instructed to find the target “T” amongst the distractors “L” and respond to its orientation (90° or 270°), that is, its left or right pointing direction, as quickly and as accurately as possible on each trial (see Fig. 1a). Depending on the target’s orientation, they had to press the corresponding (left or right) arrow key using their left- or right-hand index finger, respectively. Each trial began with a central fixation marker (0.4° × 0.4° visual angle in size) shown randomly from 800 ms to 1300 ms, which was followed by a search display. For the free-control trials, the search display was presented for 2 s and was automatically terminated either after 2 s or immediately upon the response made within the 2-s period. For the constrained-control trials, the search display duration was randomized between 800 ms and 2 s, and participants were unable to terminate the display after making a response. This random presentation time of constrained-control trials meant that participants could not anticipate when the search display would end, thereby reducing the contingency between response and display offset while avoiding a fixed temporal rhythm. In addition, the random duration of the search display in the constrained condition added a level of unpredictability, which may have further motivated participants to remain focused and responsive. No feedback was provided after the response during the training phase. The minimal duration (800 ms) of the search display (in the constrained-control trials) was chosen based on a prior contextual cueing experiment also including eight repeated displays (and new displays), with a mean reaction time of 845 ms within a 95% confidence interval (CI) ranging from 778.62 ms to 911.10 ms at the beginning of the learning (i.e., in the first block; e.g., Chen et al., 2024). For all trials, participants had to make a response within 2.1 s after the display onset; otherwise, the response was recorded as a time-out ‘error’. After a 1-s blank screen, the next trial started. Figure 1b depicts the presentation time distribution for all the Constrained Control (left) and Free Control (right) trials during the training phase. In the Constrained Control condition, presentation times are more evenly spread across the range, reflecting externally controlled display durations. In contrast, the Free Control condition shows a skewed distribution, with a higher frequency of shorter presentation times, reflecting participants’ self-paced responses. The mean presentation time for the constrained control condition (1485 ms) was significantly longer than the free-control condition (846 ms), *t* (23) = 29.32, *p* < .001, *d*_*z*_ = 5.99. Although the constrained-control manipulation resulted in longer exposure for context–target encoding, the contextual cueing effect was not expected to be modulated by this variation in presentation time. Previous studies have consistently shown that contextual cueing is robust across a wide range of display durations, emerging even under brief exposures (< 300 ms) and not being substantially enhanced by prolonged presentation times (Chaumon et al., 2008; Geyer et al., 2010; Võ & Wolfe, 2012; Xie et al., 2020).

The training phase consisted of 10 blocks: five free-control blocks and five constrained-control blocks, which were randomly interleaved. Each block contained 24 trials, organized as three repetitions of an 8-trial set. Each 8-trial repetition contained four old displays and four new displays, intermixed in random order. Thus, each block contained 12 old-display trials and 12 new-display trials. Across the training phase, each old display was repeated 15 times.

To measure the effect of our manipulation on agency experience, we obtained participants’ agency ratings at the end of each block. We used a brief single-item perceived-control rating to minimize interruption to the contextual-cueing task. Similar post-block control ratings have been used in recent agency studies to assess perceived control over action outcomes (Ren et al. 2023a, b; Giersiepen et al. 2024). The present item was intended to capture perceived control within the current block context, rather than to provide a multidimensional measure of sense of agency. Participants rated their agreement with the statement, “I was in control over my action in this block,” using a five-point scale ranging from “Fully Disagree” to “Fully Agree”. These ratings were retrospective and provided only after the search trials in each block had ended. After providing their agency ratings, participants could proceed to the next block at their own pace. To familiarize themselves with the task, participants completed a practice session consisting of blocks with 16 trials each, incorporating both free- and constrained-control trials.

During the test phase, repeated displays from both free- and constrained-control training conditions were interspersed with newly generated displays in each block. Each block consisted of 16 trials: 8 repeated displays (4 trained under the free-control condition and 4 under the constrained-control condition), and 8 new displays (4 with target locations matched to those from the free-control condition and 4 matched to those from the constrained-control condition during the training phase). For all trials, the display terminated automatically either after 3 s or immediately upon the response made within the 3-s period. A feedback message about the accuracy of the current block was presented in the middle of the screen for 500 ms at the end of each block (instead of the sense of agency rating). There were 15 blocks in the test phase. In all other respects, the experiment remained the same as in the training phase.

Thus, each participant completed 120 training trials under the free-control condition and 120 under the constrained-control condition, followed by 240 test trials conducted entirely under the free-control condition. After completing the formal visual search task, participants performed an old/new-display recognition test involving 32 trials: eight old (repeated) displays from the visual search task were repeated twice, randomly interspersed with 16 newly generated displays. Participants had to classify a given display as “old” (i.e., encountered previously in the search task) or “new” (never seen before) by pressing the left or the right arrow key, respectively.

#### Data analyses

Trials with response errors (4%) and extreme reaction times (RTs; 2.5%), either below 200 ms or above 2.5 standard deviations from an individual’s average RT per condition, were excluded from RT analysis. Notably, the mean RTs were significantly slower for incorrect responses compared to correct responses, mean difference = 210 ms, *t* (23) = 9.22, *p* < .001, *d*_*z*_ = 1.88. However, after excluding the time-out trials, the mean RTs were comparable for incorrect and correct responses, mean difference = 23 ms, *t* (23) = 0.74, *p* = .47, *d*_*z*_ = 0.15.

Contextual cueing was operationalized as faster responses to old displays than to new displays. Because our main question concerned the acquisition of contextual regularities over time, we focused not only on the mean old–new RT difference, but also on how this difference developed across epochs. Epochs were defined every 48 trials, corresponding to two trial blocks: one free-control block and one constrained-control block, ensuring that the two action-control conditions were compared over the same amount of task time throughout the experiment. Thus, contextual learning was assessed by the interaction between Display and log(Epoch), with a steeper RT reduction for old than new displays indicating the gradual emergence of contextual cueing. Critically, the influence of action-effect control on contextual learning was tested by whether this learning-related old–new difference varied between the free-control and constrained-control conditions, as reflected in the log(Epoch) × Display × Action Control interaction.

Mixed-effects modeling is well suited to this question because it retains valid trial-level observations, accommodates unequal numbers of trials after exclusion, and models subject-level variability in both baseline RTs and learning slopes. Thus, we analyzed trial-level RTs using linear mixed-effects models (LMMs) implemented in lme4 (R 4.3.3; Bates et al., 2015) under maximum likelihood. Because RT decreases were approximately linear on a logarithmic scale, Epoch was log-transformed and centred. Categorical predictors were Display (Old vs. New), Action Control (Free vs. Constrained), and Phase (Training vs. Test). Categorical predictors used treatment coding (reference levels: New display; Constrained control).

We began with a full model including all the main effects and interaction effects:

$$\:\begin{array}{l}RT\:\sim\:log\left(Epoch\right)\:\times\:\:Display\:\times\:\:\\\:Action\:Control\:\times\:\:Phase\end{array}$$

The random-effects structure included by-subject random intercepts and random slopes for all within-subject factors (Barr et al., 2013). As this structure converged reliably, we retained it throughout model comparison.

To identify the most parsimonious and appropriate fixed-effects structure, we used likelihood ratio tests (LRTs) and AIC/BIC to compare the full model to progressively simpler, hierarchically valid nested models. At each step, we removed the highest-order non-significant term and re-evaluated the model fit. This backward-elimination procedure continued until removing an additional term significantly worsened model fit (*p* < .05). For interpretability we retained main effects in all models; our a priori hypothesis concerned the learning-rate interaction *log(Epoch)*: *Display: Control*. This approach balances explanatory power and parsimony while maintaining strong Type-I error control (Bates et al., 2015). Systematic backward elimination identified a more parsimonious model that retained effects contributing meaningfully:

$$\begin{array}{l}RT\;\sim\;1\;+\;log\left(Epoch\right)\:+\:Display\;+\\\:\;Action\:Control\;+\;Phase\;+\;log\left(Epoch\right):\:\\\:\begin{array}{l}Phase\;+\;log\left(Epoch\right):Display\;+\\\:\;Display:\:Action\:Control\;+\\\:\;log\left(Epoch\right):Display:\:Action\:Control\:+\:\\\:\begin{array}{l}(1\;+\;log(Epoch)+Action\:Control\;+\;\\\:Display\;+\;Phase\:\left|\:Participant\right)\end{array}\end{array}\end{array}$$

The LRT indicated that the full model did not improve model fit relative to the reduced model (∆AIC (full-reduced) = + 3, ∆BIC (full-reduced) = + 46, *χ²* = 9.03, *df* = 6, *p* = .17), despite its substantially higher complexity. Accordingly, we retained the reduced model and moved the full model-comparison details to Supplementary Tables S1-S2 to keep the main text focused on the key effects. RT slopes (i.e., the change in RT across log-transformed epochs) were estimated for each condition using the emtrends function in the emmeans package.

To analyze trial-wise accuracy, we employed a generalized linear mixed-effects model (GLMM) with a binomial link function. Model selection followed a similar procedure to the RT analysis (see Table S3 in supplementary materials). To prevent convergence issues, the random-slopes structure was limited to a subset of factors. The final model was specified as follows:

$$\begin{array}{l}Accuracy\;\sim\;1\;+\;log\left(Epoch\right)+\;\\\:Action\:Control+Display\;+\;Phase+\;\\\begin{array}{l}log\left(Epoch\right):\:Display\;+\\log\left(Epoch\right):\:Action\:Control\;+\\\begin{array}{l}Action\:Control:\:Phase\:+\\\begin{array}{l}(1\;+\;log(Epoch)+Display\:+\\Phase\:\left|\:Participant\right)\end{array}\end{array}\end{array}\end{array}$$

The LRT also indicated that the full model did not improve model fit relative to the reduced model (∆AIC (full-reduced) = + 8, ∆BIC (full-reduced) = + 66, *χ²* = 8.44, *df* = 8, *p* = .39). Effect estimates are reported as odds ratios (OR). An odds ratio (OR) > 1 means the odds of the event (e.g., a correct response) increase with the predictor, while an OR < 1 means the odds of the event decrease with the predictor. Complete results are provided in Supplementary Table S4.

## Results

### Response times

**Fig. 2:**
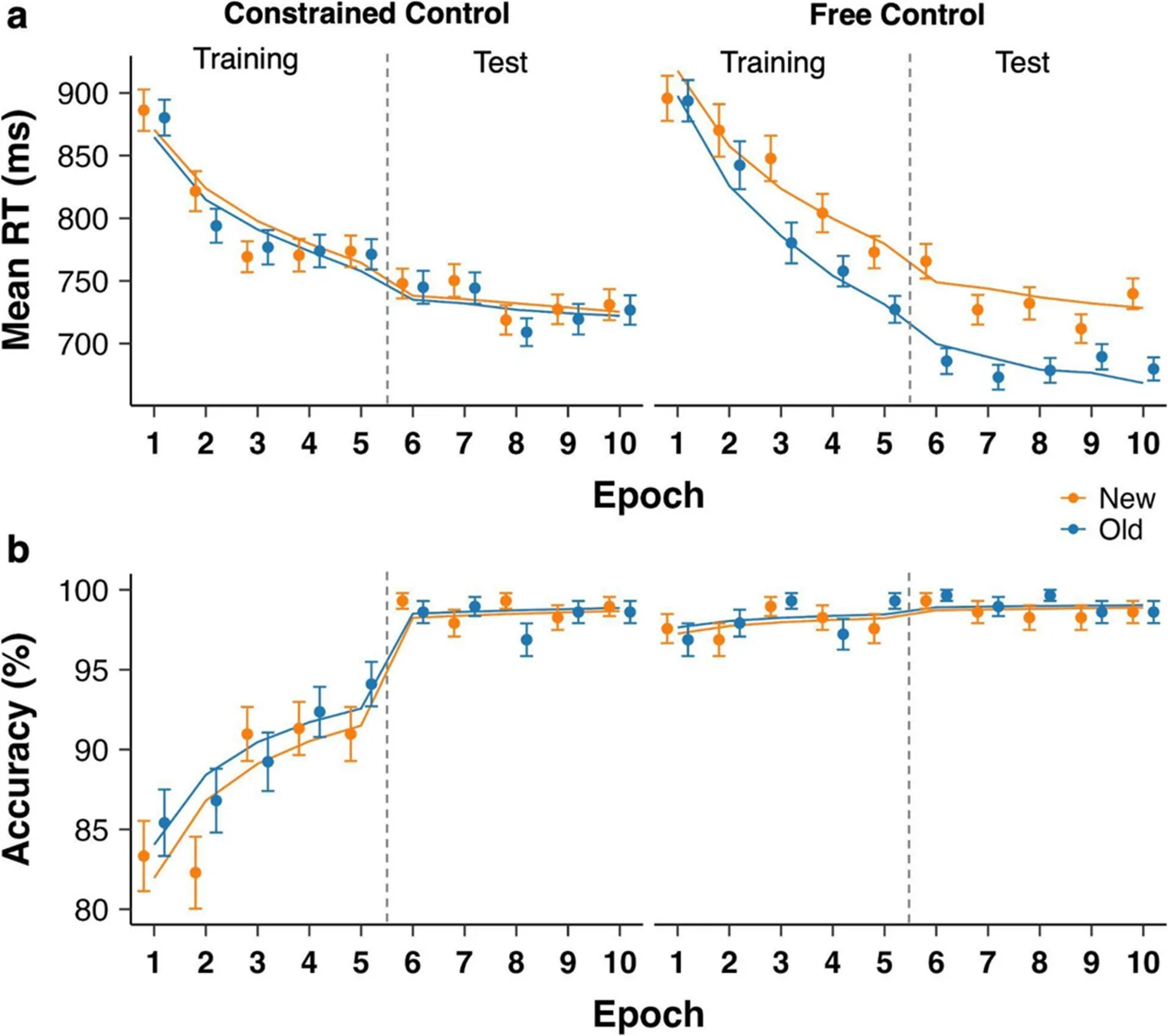
Mean RTs (**a**) and accuracies (**b**) observed (dots) and RT/accuracy estimated by the LMMs/GLMMs (lines) for the old (blue) and new (orange) displays separately in free-control and constrained-control conditions as a function of Epoch number (one epoch averaging 48 trials) in the training and test phases (with the transition marked by a dashed vertical line). Error bars represent within-participant SEMs. The constrained- and free-control panels use the same y-axis scale to allow direct comparison between sessions

Figure 2a presents the observed RTs (indicated by the dots with associated error bars) and the predicted RTs (indicated by the curves) from the LMM. RTs decreased reliably with log-transformed epoch in the training phase (*β* = -71.8 ms, 95% CI [-92.4, -51.1], *p* < .001; intercept = 772 ms [739, 805], *p* < .001). A significant Phase × log(Epoch) interaction, *β* = 45.2 [13.8, 76.7], *p* = .005, indicated that the RT slope across epochs differed between training and test. Specifically, this interaction tests whether the decrease in RT over epochs was larger in one phase than in the other. Estimated simple slopes clarified this interaction: RTs decreased significantly across epochs during training, *β* = -83.0 ms [-100.9, -65.0], and also during test, *β* = -37.7 ms [-71.2, -4.3]. Thus, the RT reduction across epochs was steeper during training than during test. The Display × Control interaction (*β* = -41.4 [-56.9, -25.9], *p* < .001) revealed faster responses to old displays in the free-control condition (*M* = 726 ms [693, 759]) than the constrained-control condition (*M* = 753 ms [720, 786], *p* = .003). In contrast, mean RTs for new displays did not differ reliably between free-control (*M* = 773 ms [739, 807]) and constrained-control conditions (*M* = 759 ms [726, 792], *p* = .13). Critically, RT slopes for old displays were substantially steeper in the free-control condition (*M* = -80.9 ms [-104.7, -57.2]) than in the constrained-control condition (*M* = -46.9 ms [-70.8, -23.1], *p* < .001). For new displays, RT slopes did not differ significantly between free-control (*M* = -64.4 ms [-88.1, -40.6]) and constrained-control conditions (*M* = -49.1 ms [-73.1, -25.2], *p* = .25). These results provided evidence for the development of contextual cueing (RT_new_ - RT_old_ = 47.16 ms, *p* < .001) in the Free-Control condition, but not in the Constrained-Control condition (RT_new_ - RT_old_ = 5.76 ms, *p* = .52).

### Accuracy

 The GLMM revealed a significant positive effect of log(Epoch), indicating that accuracy improved over time (*OR* = 1.70 [1.29, 2.23], *p* < .001; see Fig. 2b). There was an effect of Phase (*OR* = 0.21 [0.13, 0.36], *p* < .001), with lower accuracy in the training phase (96.7% [95.4%, 97.6%]) than in the test phase (98.6%, [98%, 99.1%]). Critically, a significant interaction between Phase and Action control (*OR* = 3.59 [1.64, 7.86], *p* < .001) indicated that accuracy was significantly lower in training than test phases in the constrained-control condition (92.6% [90.2%, 94.4%] vs. 98.3% [97.5%, 98.9%], *p* < .001) but remained high and comparable between phases in the free-control condition (98.5% [97.7%, 99.1%] vs. 98.9% [98.1%, 99.3%], *p* = .90).

### Agency experience

**Fig. 3:**
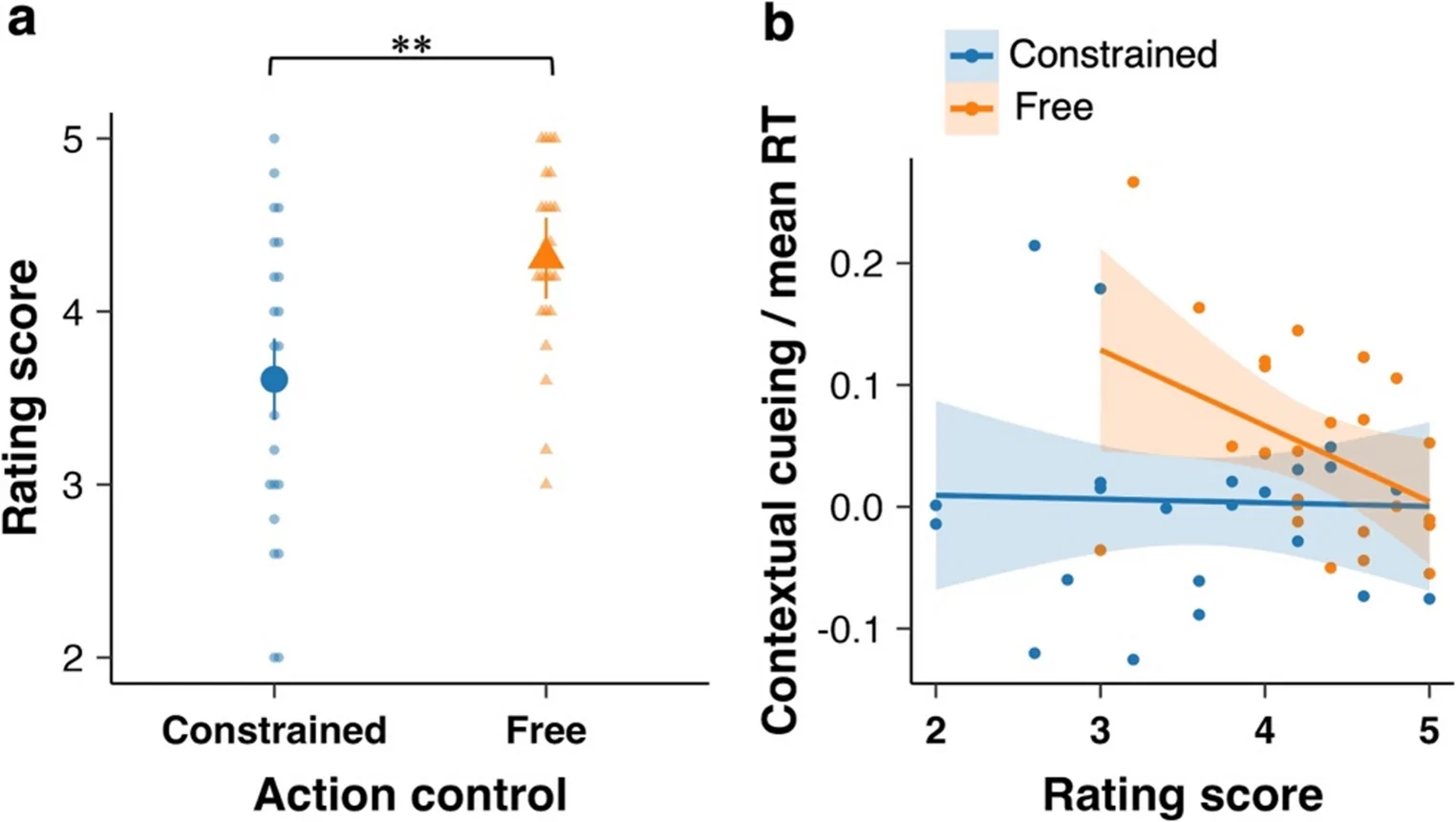
**a** Rating of sense of agency (SoA) in the training phase. Error bars represent within-participant SEMs. ** indicates *p* < .001. **b** Relationship between perceived-control ratings and RT-normalized contextual cueing, calculated as (RT_New_−RT_Old_)/RT_mean_, separately for the Constrained-Control and Free-Control conditions. Each dot represents a participant, and colored lines indicate the best-fitting linear regression for each control condition. Shaded regions represent 95% confidence intervals

The results of subjective evaluation during the training phase (Fig. 3a) revealed a significantly higher rating score of SoA (*M* = 4.31) for free than constrained-control conditions (*M* = 3.61), *t* (23) = 4.36, *p* < .001, *d*_*z*_ = 0.89. To examine the relationship between subjective ratings and contextual cueing, we calculated a cueing score for each participant and condition as the mean RT for new displays minus the mean RT for old displays, with larger positive values indicating stronger contextual cueing. To reduce the influence of individual differences in overall response speed, contextual cueing was expressed as a relative score, (RT_New_−RT_Old_) / RT_mean_. In the Free-Control condition, higher perceived-control ratings were associated with smaller relative contextual-cueing effects, *b* = − 0.062, SE = 0.028, *t* (22) = − 2.19, *p* = .039, *R*^*2*^ = 0.179. In the Constrained-Control condition, perceived-control ratings were not associated with relative contextual cueing, *b = −* 0.003, SE = 0.021, *t* (22) = − 0.15, *p* = .885, *R*^*2*^ = 0.001. A robust regression in the Free-Control condition yielded a comparable negative estimate, *b* = − 0.071, SE = 0.027, *t = −* 2.59, suggesting that the association was not solely driven by extreme observations.

This inverse association was unexpected and does not support a straightforward positive relationship between subjective perceived control and contextual learning. One possibility is that participants who experienced the Free-Control task as more fluent or controllable were already relatively efficient on novel displays and therefore showed less additional benefit from repeated configurations. Conversely, participants with lower perceived-control ratings may have relied more strongly on learned contextual information, producing larger contextual cueing. However, this interpretation remains speculative. The association was exploratory, based on a small sample, and estimated between participants. Participants may also have interpreted the rating question more broadly as referring to perceived control over the task or over the action outcome. Thus, the rating measure should be viewed as an index of subjective perceived control in the task context, rather than as a process-pure measure of sense of agency. Taken together, the rating data do not provide convergent evidence that stronger subjective agency enhanced contextual learning, and we therefore refrain from assigning the inverse association a mechanistic interpretation.

### Recognition results

Participants’ explicit recognition performance – that is, their ability to tell apart repeated displays (‘signals’) from non-repeated displays (‘noise’) – was assessed by the signal-detection sensitivity parameter *d’* (z[hits] – z[false alarms]; Green & Swets, 1966), treating correct recognition of repeated displays as ‘hits’ and incorrect ‘recognition’ of novel displays as ‘false alarms’. The mean *d’* score (*M* = 0.23) turned out greater than zero for free-control trials, *t* (23) = 2.13, *p* = .04, *d* = 0.43 – consistent with Chen et al.’s (2024) findings using four repeated displays showing that participants had learned to explicitly recognize (at least some of) repeated displays during the search task. In contrast, for constrained-control trials, *d’* was relatively small, 0.14, and failed to differ significantly from zero, *t* (23) = 1.18, *p* = .25, *d* = 0.24. This suggests that observers were largely unaware of the repetition of (specific) old-context displays when they could not freely control the outcome of their response.

## Experiment 2

### Methods

Thirty participants took part in Experiment 2 (13 men; *M* = 25.8, 28 right-handed) with normal or corrected-to-normal visual acuity. This experiment adopted a 2 (Outcome Presence: absent vs. present) × 2 (Outcome Probability for Old Configurations: high vs. low) within-subjects design. The factor “Outcome Presence” was manipulated at the trial-by-trial level, and outcome probability was manipulated at the session level. We used a “correct”/“error” feedback as the action outcomes that are frequently used in contextual cueing research (e.g., Chen et al. 2020, 2022a, b a, Chen et al. 2022a, b b). Each block comprised four old and four novel spatial configurations. In Session A (Old-High, New-Low), three of the four old configurations were followed by performance-contingent feedback (i.e., outcome trials), while one was followed by no feedback. Conversely, only one of the four new configurations was followed by feedback, with the remaining three followed by no outcome. In Session B (Old-Low, New-High), this contingency structure was reversed: one old configuration was followed by feedback, and three by no feedback. For new configurations, three were followed by feedback, and one by no feedback. That is, when the probability of outcome presence for old configurations was high (75%), it was low (25%) for new configurations, and vice versa (see Table 1). The choice of probability values (75% vs. 25%) was based on previous work (Ren et al. 2023a), which demonstrated that such a manipulation reliably modulates perceived agency through variations in outcome presence. The order of the Old-High/New-Low and Old-Low/New-High sessions was counterbalanced across participants.

**Table 1:** Experimental design

	Experimental Manipulation		Outcome Presence (trial-wise)	
			Absent	Present
Outcome Probability	**Session A**	New-Low	75% trials	25% trials
		Old-High	25% trials	75% trials
(Block-wise)	**Session B**	New-High	25% trials	75% trials
		Old-Low	75% trials	25% trials

Each trial began with a fixation display presented for a variable interval of approximately 800–1300 ms. The search display then appeared and remained on screen until the participant responded, or until a 3000-ms response deadline was reached. Thus, Experiment 2 used the free-control trial structure from Experiment 1: the participant’s response terminated the search display. After display offset, a blank screen was presented for a randomly selected interval of up to 500 ms. On feedback-present trials, performance feedback (“Correct!” or “Error!”) was then presented for 800 ms. On feedback-absent trials, no performance feedback was shown; instead, a fixation display was presented for the same 800-ms duration. Thus, the post-response interval was matched between feedback-present and feedback-absent trials. The next trial then began with the fixation display. Different old configurations were used in the two sessions. One session consists of 256 trials, with each old display repeating 32 times (32 blocks). Old and new displays were intermixed randomly in each block. In addition, participants were also required to rate how much control they felt over the task outcome on a five-point scale ranging from “Fully Disagree” to “Fully Agree” every 4 blocks. After providing their agency ratings, participants could proceed to the next block at their own pace. In total, participants rated their sense of agency 8 times in each session. To familiarize themselves with the task, participants completed a practice session consisting of two blocks with random outcome probabilities at the beginning. At the end of the experiment, participants performed an old/new-display recognition test involving 32 trials: eight old (repeated) displays from both sessions were repeated twice, randomly interspersed with 16 newly generated displays.

Trials with response errors (1%) and extreme reaction times (RTs; 2.5%), either below 200 ms or above 2.5 standard deviations from an individual’s average RT per condition, were excluded from RT analysis. We analyzed trial-level RTs using linear mixed-effects models (LMMs). We created a new factor “Epoch” for every 32 trials, comprising four consecutive trial blocks. The key predictors were log(Epoch), Display (Old vs. New), Outcome Presence in the previous trial (absent vs. present), and Session (Session A, Session B). The random-effects structure included by-subject random intercepts and random slopes for all within-subject factors.

Model selection followed a similar procedure to Experiment 1 by comparing the full model to progressively simpler, hierarchically valid nested models. The final model was specified as follows:

$$\:\begin{array}{l}RT\;\sim\;1\;+\;log\left(Epoch\right)\:+\:Display\;+Session\;+\;\\Outcome\:Presence\;+\;log\left(Epoch\right):\:Display\;+\\\begin{array}{l}Display:\:Session\:+\:(1\;+\;log(Epoch)\:+Display\;+\\Session\;+\;Outcome\:Presence\:\left|\:Participant\right)\end{array}\:\end{array}$$

The LRT indicated that the full model did not improve model fit relative to the reduced model (ΔAIC (full-reduced) = + 12, ΔBIC (full-reduced) = + 81, *χ²* = 5.79, *df* = 9, *p* = .76). Model-comparison statistics and results are reported in Supplementary Tables S5 and S6.

Mean accuracies were high across all conditions (98.4% ~ 99.1%) with no apparent differences, so we did not model accuracy further.

## Results

### Response times

**Fig. 4:**
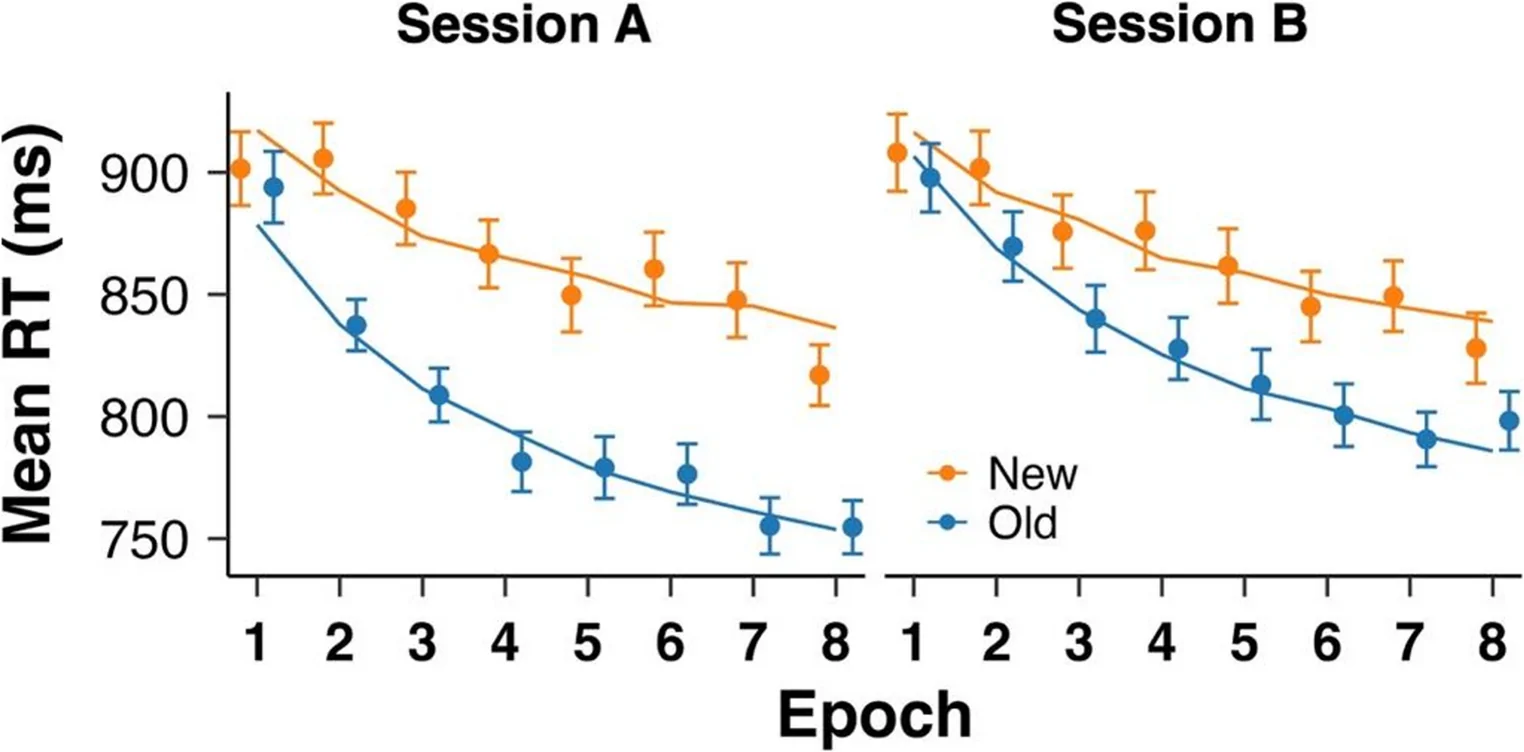
Mean RT observed (dots) and RT estimated by the LMMs (lines) for the old (blue) and new (orange) displays separately in Session A (Old-High, New-Low) and Session B (Old-Low, New-High) as a function of Epoch number (one epoch averaging 32 trials). The two panels use the same y-axis scale to allow direct comparison between sessions

Figure 4 presents the observed RTs (indicated by the dots with associated error bars) and the predicted RTs (indicated by the curves) from the LMM. The LMM showed clear evidence of learning: RTs decreased reliably with log-transformed epoch (*β* = -37.5 ms [-51.4, -23.5], *p* < .001; intercept = 871 ms [805, 938], *p* < .001). Old displays elicited faster responses than new displays (*β* = -37.4 ms [-58.3, -16.5], *p* < .001), and this benefit increased over time, as indicated by a steeper learning slope for old than new displays (*β* = -22.4 ms [-34.1, -10.7], *p* < .001). Trials preceded by outcome were faster than those preceded by no outcome (*β* = -10.2 ms [-18.9, -1.4], *p* = .023). Although mean RTs were comparable between sessions (*p* = .98), the contextual-cueing effect was substantially larger in Session A than in Session B (*β* = -31.7 ms [-47.0, -16.5], *p* < .001).

### Agency experience

The results of subjective evaluation revealed no significant difference in agency ratings between the two sessions with high (*M* = 4.48) and low (*M* = 4.34) outcome probability, *t* (29) = 1.57, *p* = .13, *d*_*z*_ = 0.29. Furthermore, separate linear regressions indicated no significant relationships between rating scores and the contextual cueing effect in either session (*p*s > 0.41). This lack of difference may reflect that the overall frequency of outcome feedback was equal across the two sessions. Moreover, subjective agency ratings were collected once per block and thus necessarily averaged across old and new configurations. This block-level measure is likely too coarse-grained to capture subtle, configuration-specific differences in action-outcome contingencies.

### Recognition results

Participants’ explicit recognition performance, measured by signal-detection sensitivity (*d′*), indicated an ability to distinguish repeated displays (“signals”) from non-repeated displays (“noise”). The mean *d′* was greater than zero in both sessions with high (*M* = 0.43, *t*(29) = 3.26, *p* = .003, *d* = 0.60) and low (*M* = 0.46, *t*(29) = 3.22, *p* = .003, *d* = 0.59) outcome probabilities. Recognition sensitivity was above chance in both sessions and was numerically comparable for old configurations associated with high versus low outcome probability. Thus, participants showed some explicit awareness of repeated configurations, but this awareness did not appear to differ systematically as a function of outcome probability. The larger contextual-cueing effect in the Old-High session is therefore unlikely to be explained solely by stronger explicit old/new discrimination.

## Discussion

The present study examined whether agency-related processes influence contextual learning. We focused on two components that are highlighted in hierarchical models of agency: action-effect control, reflecting the reliability with which one’s actions produce immediate perceptual consequences (Experiment 1), and outcome-based agency cues, reflecting configuration-specific contingencies between responses and performance outcome (Experiment 2). Both experiments are consistent with prior research demonstrating robust contextual cueing effects under voluntary control (Chen et al., 2024; Chun & Jiang, 1998; Sisk et al., 2019). Importantly, we found that reducing action-effect control during the learning phase impaired the development of contextual cueing effects even after full control was restored during the test phase. Moreover, contextual learning was selectively enhanced when old configurations were more reliably followed by outcome. Together, these findings suggest that reliable response–effect relations—an important component of experienced agency—may support the acquisition of spatial regularities.

For Experiment 1, we predicted that if SoA plays a role in the initial encoding of contextual information, then limiting action-effect control during the learning phase (i.e., constrained-control conditions) should produce lasting impairments in contextual learning, even when full control is later restored. In contrast, if SoA primarily affects the retrieval or expression of learned information, then any contextual learning deficits should resolve once autonomy is reinstated in the test phase. Consistent with the encoding-based hypothesis, we observed a contextual cueing effect (i.e., faster RT reductions for old vs. new displays) under the free-control condition rather than the constrained-control condition, indicating that higher agency during training facilitated the acquisition of contextual information. Importantly, the lack of phase-related improvement suggests that impairments observed in the constrained-control condition did not recover even after full control was restored in the test phase. This suggests that externally imposed limitations on action-effect control interfere with the early encoding stages rather than just behavioral expression. The advantage of free control in contextual learning emerged despite the fact that configurations were displayed for a longer duration in the constrained-control condition. Notably, such extended exposure would ordinarily be expected to enhance stimulus encoding; however, prior studies have consistently shown that contextual cueing is largely unaffected by variations in display duration (Geyer et al., 2010; Võ & Wolfe, 2012; Xie et al., 2020). These findings suggest that when participants could freely determine when the display disappeared, the response–offset relation created a more reliable action-effect context, which may have supported engagement with the search episode and facilitated the use of contextual cues.

Of note, contextual cueing for the previously constrained configurations did not clearly emerge even after full response-dependent control was restored in the Test phase. This suggests that these configurations were not readily learned under the new Free-Control conditions, despite now being presented with the same response–offset contingency as the initially Free-Control configurations. The Constrained-Control manipulation involved longer and less predictable display durations, a variable interval between the response and display offset, and different opportunities to encode associations among repeated configurations, response timing, and display duration. When action-effect control is reduced, participants may have formed general associations between the Constrained-Control context and characteristic response timing for both old and new displays. In addition, because old configurations were repeatedly encountered, they may have acquired more specific associations with response latency or externally determined display offset. Such prior learning could have interfered with the subsequent acquisition and expression of contextual cueing once free control was restored. The present design cannot determine whether the subsequent absence of contextual cueing reflects interference from previously acquired stimulus–response timing associations, limited relearning after control was restored, or a combination of these processes.

The condition-level rating data confirmed that the manipulation affected participants’ subjective experience of control: perceived-control ratings were higher in the Free-Control than in the Constrained-Control condition. This finding is consistent with the intended effect of the manipulation on the broader action–outcome context. However, the individual-differences analysis did not provide convergent evidence that participants who experienced greater control also showed stronger contextual learning. Instead, within the Free-Control condition, higher perceived-control ratings were associated with smaller relative contextual-cueing effects. However, the analysis was exploratory, based on a small sample, and estimated across participants. The association may therefore reflect stable individual differences in response fluency, search efficiency, confidence, task engagement, or rating style rather than a direct relationship between perceived control and contextual learning. In addition, participants may have interpreted the rating item broadly as referring to control over the task, rather than to a process-pure sense of agency. Accordingly, the rating measure should be regarded as an index of subjective perceived control within the task context. More generally, the condition-level effect and the individual-differences association suggest that experimentally induced differences in perceived control and variation in contextual-cueing performance across individuals need not vary in parallel.

Experiment 2 manipulated outcome-based agency cues by making feedback more likely after responses to either old or new displays in separate sessions, while keeping the overall feedback rate constant across sessions. Because the outcome contingency was reversed across sessions and overall outcome frequency was matched, the larger contextual-cueing effect in the Old-High/New-Low session cannot be explained by a global increase in outcome frequency. Rather, the result suggests that contextual learning was strengthened when repeated configurations were more reliably linked to action outcomes. Given that feedback frequency was matched across sessions, a purely motivational account is unlikely. The modest speeding following an outcome on the immediately preceding trial did not vary with epoch or display type, suggesting that the larger contextual-cueing effect in Session A cannot be explained solely by immediate, nonspecific trial-to-trial facilitation (cf. Chun & Jiang, 1998). Instead, the data suggest that observers integrated the global contingency structure over many trials and assigned greater weight to contexts that more reliably predicted action consequences, thereby updating context–target associations and biasing attention. Thus, a single feedback event may have been too weak or noisy to update spatial associations effectively; instead, the cognitive system appeared to rely on the accumulation of consistent action-outcome patterns to infer a stable sense of control and influence learning (Oishi et al., 2019). This cumulative integration aligns with hierarchical predictive-coding accounts, in which higher-level control states emerge from the aggregation of lower-level prediction outcomes across extended time windows (Friston, 2010). Moreover, the temporal alignment of actions and salient outcome may further enhance memory through mechanisms such as event binding or contextual integration, whereby actions and their outcomes become more tightly coupled in memory (Hommel, 2009; Waszak et al., 2012). Importantly, the Experiment 2 effect should not be interpreted as showing that action-outcome contingency is necessary for contextual learning. Contextual cueing was still present when old configurations were rarely followed by feedback, and Experiment 1 showed that contextual cueing can emerge in the absence of performance feedback during training. Rather, action-outcome contingency modulated the strength of contextual learning.

Notably, subjective agency ratings did not differ significantly between the two sessions. This null result is not unexpected. Participants’ ratings were collected at the block level and thus averaged across old and new configurations. The behavioral contextual cueing effect was nevertheless selectively modulated by the contingency structure for old displays, indicating that participants were sensitive to these asymmetries even when they did not report overt differences in agency. Moreover, the rating item may have been especially insensitive because participants retained full control over their responses and display offset in both sessions; only the probability of receiving feedback differed between old and new displays. Therefore, the absence of a session difference in ratings does not rule out the possibility that outcome contingencies influenced learning without producing a clear conscious difference in perceived control.

Overall, our findings align well with hierarchical models of agency, which propose that learning and action are supported by multiple interacting levels of control. In this framework, higher-level volitional processes facilitate intentional exploration and the encoding of contextual information, while lower-level outcome monitoring mechanisms calibrate behavior by detecting and correcting discrepancies between expected and actual outcomes (Haggard, 2017; Synofzik et al., 2008). Specifically, contextual learning is sensitive to the reliability of action-linked perceptual consequences, highlighting the potential role of a stable action–outcome context in forming durable memory representations (see also Hon & Yeo, 2021; Zou et al., 2023). Post-response visual outcome likely engages prediction error mechanisms, driving the updating and refinement of cognitive representations. These mechanisms enable adaptive learning by continuously calibrating expectations and strengthening the mapping between spatial context and target locations.

Although our findings clarify how agency-related action and outcome processes shape contextual learning, several limitations remain. First, asking participants to rate their sense of agency during the experiment may have encouraged agency monitoring and, in principle, influenced attentional allocation or metacognitive access to the task structure. The ratings were retrospective and collected only at block boundaries, and therefore did not impose a trial-wise dual-task demand. Nevertheless, we interpret the findings cautiously as showing that agency-related manipulations influenced contextual learning under conditions in which agency was explicitly monitored. This concern is unlikely to fully account for the observed pattern, because in Experiment 1 both action-control conditions were rated, yet contextual cueing differed between them, and in Experiment 2 agency ratings did not differ across sessions, even though contextual cueing did. Second, the constrained-control manipulation in Experiment 1 reduced action-effect control, but may also have affected trial fluency, temporal predictability, post-response engagement, and adaptation to externally determined display timing. These factors are theoretically related to agency, because SoA depends partly on reliable and temporally predictable action–effect relations, and fluent action-outcome processing may support outcome monitoring and learning (Frith et al., 2000; Sato & Yasuda, 2005; Haggard, 2017; Synofzik et al., 2008; Hommel, 2009; Waszak et al., 2012). Although display offsets in the constrained-control condition were randomized and therefore did not impose a fixed temporal rhythm, participants may still have formed expectations about the average duration or temporal distribution of constrained-control trials. Thus, the present design cannot determine which component of agency-related action-effect control was most critical, or whether the effect partly reflected adaptation to externally determined display timing. Future work should use a duration-matched control design, for example by yoking display durations across Free- and Constrained-Control trials, so that response–offset contingency can be manipulated independently of average display duration, temporal variability, and post-response display persistence. This would help determine whether the present effect reflects reduced response–offset contingency, increased temporal uncertainty, adaptation to externally controlled display timing, or some combination of these factors. Third, the recognition findings require a nuanced interpretation. Contextual cueing is often predominantly implicit, but partial explicit awareness can emerge under some task settings, especially when relatively few repeated configurations are used (Chen et al. 2025a, b). We therefore do not interpret above-chance recognition as evidence against contextual cueing, but rather as evidence that the present task allowed some learned regularities to become consciously accessible. Importantly, learning remained incidental because participants were never instructed to memorize the displays. Finally, the T/L visual-search task, while well controlled, may limit ecological validity. Future work should examine whether agency-related modulation of contextual learning extends to more naturalistic search environments, including real-world or immersive virtual settings.

## Supplementary Information

Below is the link to the electronic supplementary material.

Supplementary Material 1

## Data Availability

The data and materials have been made available via the Open Science Framework and can be accessed at: [https://osf.io/dfah5](https:/osf.io/dfah5).

## References

[CR1] Barr, D. J. (2013). Random effects structure for testing interactions in linear mixed-effects models. *Frontiers in Psychology*, *4*, 328. 10.3389/fpsyg.2013.00328 PMC3672519 23761778

[CR2] Bates, D., Mächler, M., Bolker, B., & Walker, S. (2015). Fitting linear mixed-effects models using lme4. *Journal of statistical software*, *67*, 1–48. 10.18637/JSS.V067.I01

[CR3] Brainard, D. H. (1997). The Psychophysics Toolbox. *Spatial Vision*, *10*(4), 433–436. 10.1163/156856897x00357 9176952

[CR4] Chaumon, M., Drouet, V., & Tallon-Baudry, C. (2008). Unconscious associative memory affects visual processing before 100 ms. *Journal of Vision*, *8*(3), 101–110. 10.1167/8.3.10 18484816

[CR5] Chen, S., Allenmark, F., Merkuš, N., Müller, H. J., & Shi, Z. (2025a). Context-based guidance versus context suppression in contextual learning: Role of un-/certainty in the target-context relations in visual search. *Journal of Experimental Psychology Human Perception and Performance*. 10.1037/xhp0001321 40126583

[CR6] Chen, S., Geyer, T., Zinchenko, A., Müller, H., & Shi, Z. (2022a). Multisensory rather than unisensory representations contribute to statistical context learning in tactile search. *Journal of Cognitive Neuroscience*, *34*(9), 1702–1717. 10.1162/jocn_a_01880 35704553

[CR7] Chen, S., Merkuš, N., Tsai, S. Y., Cheng, S., Müller, H. J., & Shi, Z. (2025b). Statistical context learning in visual search: distinct electrophysiological signatures of contextual guidance and context suppression. *Journal of Neuroscience*, *45*(22), e2186242025. 10.1523/JNEUROSCI.2186-24.2025 PMC12121704 40246526

[CR8] Chen, S., Müller, H. J., & Shi, Z. (2024). Contextual facilitation: Separable roles of contextual guidance and context suppression in visual search. *Psychonomic Bulletin & Review*, 1–9. 10.3758/s13423-024-02508-1 PMC11680642 38689187

[CR9] Chen, S., Shi, Z., Müller, H. J., & Geyer, T. (2021). Multisensory visuo-tactile context learning enhances the guidance of unisensory visual search. *Scientific Reports*, *11*(1), 9439. 10.1038/s41598-021-88946-6 PMC8093296 33941832

[CR10] Chen, S., Shi, Z., Zang, X., Zhu, X., Assumpção, L., Müller, H. J., & Geyer, T. (2020). Crossmodal learning of target-context associations: When would tactile context predict visual search? *Attention Perception & Psychophysics*, *82*(4), 1682–1694. 10.3758/s13414-019-01907-0 PMC7297845 31845105

[CR11] Chen, S., Shi, Z., Zinchenko, A., Müller, H. J., & Geyer, T. (2022b). Cross-modal contextual memory guides selective attention in visual-search tasks. *Psychophysiology*, *59*(7), e14025. 10.1111/psyp.14025 35141899

[CR12] Chun, M. M., & Jiang, Y. (1998). Contextual cueing: implicit learning and memory of visual context guides spatial attention. *Cognitive Psychology*, *36*(1), 28–71. 10.1006/cogp.1998.0681 9679076

[CR13] Friston, K. (2010). The free-energy principle: a unified brain theory? *Nature Reviews Neuroscience*, *11*(2), 127–138. 10.1038/nrn2787 20068583

[CR14] Frith, C. D., Blakemore, S. J., & Wolpert, D. M. (2000). Abnormalities in the awareness and control of action. *Philosophical Transactions of the Royal Society of London Series B: Biological Sciences*, *355*(1404), 1771–1788. 10.1098/rstb.2000.0734 PMC1692910 11205340

[CR15] Geng, J. J., & Behrmann, M. (2005). Spatial probability as an attentional cue in visual search. *Perception & Psychophysics*, *67*(7), 1252–1268. 10.3758/bf03193557 16502846

[CR16] Geyer, T., Zehetleitner, M., & Müller, H. J. (2010). Contextual cueing of pop-out visual search: when context guides the deployment of attention. *Journal of Vision*, *10*(5), 20. 10.1167/10.5.20 20616131

[CR17] Giersiepen, M., Schütz-Bosbach, S., & Kaiser, J. (2024). My choice, my actions: self-determination, not instrumental value of outcomes enhances outcome monitoring during learning. *Cerebral Cortex*, *34*(8), bhae325. 10.1093/cercor/bhae325 39118215

[CR18] Green, D. M., & Swets, J. A. (1966). *Signal detection theory and psychophysics*. Wiley.

[CR20] Haggard, P. (2017). Sense of agency in the human brain. *Nature Reviews Neuroscience*, *18*(4), 196–207. 10.1038/nrn.2017.14 28251993

[CR21] Hommel, B. (2009). Action control according to TEC (theory of event coding). *Psychological Research Psychologische Forschung*, *73*(4), 512–526. 10.1007/s00426-009-0234-2 PMC2694931 19337749

[CR23] Hon, N., & Yeo, N. (2021). Having a sense of agency can improve memory. *Psychonomic Bulletin & Review*, *28*(3), 946–952. 10.3758/s13423-020-01849-x 33415660

[CR24] Hout, M. C., & Goldinger, S. D. (2012). Incidental learning speeds visual search by lowering response thresholds, not by improving efficiency: evidence from eye movements. *Journal of Experimental Psychology Human Perception and Performance*, *38*(1), 90–112. 10.1037/a0023894 PMC3158272 21574743

[CR25] Huffman, G., & Brockmole, J. R. (2020). Attentional selection is biased towards controllable stimuli. *Attention Perception & Psychophysics*, *82*(5), 2558–2569. 10.3758/s13414-020-02004-3 32166643

[CR26] Johnson, J. S., Woodman, G. F., Braun, E., & Luck, S. J. (2007). Implicit memory influences the allocation of attention in visual cortex. *Psychonomic Bulletin & Review*, *14*(5), 834–839. 10.3758/bf03194108 18087946

[CR27] Kunar, M. A., Flusberg, S., Horowitz, T. S., & Wolfe, J. M. (2007). Does contextual cuing guide the deployment of attention? *Journal of Experimental Psychology Human Perception and Performance*, *33*(4), 816–828. 10.1037/0096-1523.33.4.816 PMC2922990 17683230

[CR28] Pelli, D. G. (1997). The VideoToolbox software for visual psychophysics: transforming numbers into movies. *Spatial Vision*, *10*(4), 437–442. 10.1163/156856897X00366 9176953

[CR45] Oishi, H., Tanaka, K., & Watanabe, K. (2019). Sense of agency in continuous action is influenced by outcome feedback in one-back trials. *Acta Psychologica*, *199*, 102897. 10.1016/j.actpsy.2019.102897 31365896

[CR29] Ren, Q., Gentsch, A., Kaiser, J., & Schütz-Bosbach, S. (2023a). Ready to go: Higher sense of agency enhances action readiness and reduces response inhibition. *Cognition*, *237*(105456), 105456. 10.1016/j.cognition.2023.105456 37037164

[CR30] Ren, Q., Kaiser, J., Gentsch, A., & Schütz-Bosbach, S. (2023b). Prepared to stop: how sense of agency in a preceding trial modulates inhibitory control in the current trial. *Cerebral Cortex*, *33*(13), 8565–8580. 10.1093/cercor/bhad141 37125462

[CR31] Sato, A., & Yasuda, A. (2005). Illusion of sense of self-agency: discrepancy between the predicted and actual sensory consequences of actions modulates the sense of self-agency, but not the sense of self-ownership. *Cognition*, *94*(3), 241–255. 10.1016/j.cognition.2004.04.003 15617673

[CR34] Sisk, C. A., Remington, R. W., & Jiang, Y. V. (2019). Mechanisms of contextual cueing: A tutorial review. *Attention Perception & Psychophysics*, *81*(8), 2571–2589. 10.3758/s13414-019-01832-2 31410759

[CR35] Synofzik, M., Vosgerau, G., & Newen, A. (2008). Beyond the comparator model: a multifactorial two-step account of agency. *Consciousness and Cognition*, *17*(1), 219–239. 10.1016/j.concog.2007.03.010 17482480

[CR36] Vadillo, M. A., Giménez-Fernández, T., Beesley, T., Shanks, D. R., & Luque, D. (2021). There is more to contextual cuing than meets the eye: Improving visual search without attentional guidance toward predictable target locations. *Journal of Experimental Psychology Human Perception and Performance*, *47*(1), 116–120. 10.1037/xhp0000780 33180547

[CR37] Võ, M. L. H., & Wolfe, J. M. (2012). When does repeated search in scenes involve memory? Looking *at* versus looking *for* objects in scenes. *Journal of Experimental Psychology: Human Perception and Performance*, *38*(1), 23–41. 10.1037/a0024147 PMC3969238 21688939

[CR38] Waszak, F., Cardoso-Leite, P., & Hughes, G. (2012). Action effect anticipation: neurophysiological basis and functional consequences. *Neuroscience and Biobehavioral Reviews*, *36*(2), 943–959. 10.1016/j.neubiorev.2011.11.004 22108008

[CR39] Wen, W., & Haggard, P. (2018). Control changes the way we look at the world. *Journal of cognitive neuroscience*, *30*(4), 603–619. 10.1162/jocn_a_01226 29308984

[CR41] Xie, X., Chen, S., & Zang, X. (2020). Contextual cueing effect under rapid presentation. *Frontiers in Psychology*, *11*, 603520. 10.3389/fpsyg.2020.603520 PMC7793704 33424716

[CR43] Zinchenko, A., Conci, M., Töllner, T., Müller, H. J., & Geyer, T. (2020). Automatic Guidance (and Misguidance) of Visuospatial Attention by Acquired Scene Memory: Evidence From an N1pc Polarity Reversal. *Psychological Science*, *31*(12), 1531–1543. 10.1177/0956797620954815 PMC7734553 33119432

[CR44] Zou, X., Chen, Y., Xiao, Y., Zhou, Q., & Zhang, X. (2023). Better controlled, better maintained: Sense of agency facilitates working memory. *Consciousness and Cognition*, *110*, 103501. 10.1016/j.concog.2023.103501 36989863

