# Supplementary material for: Sense of agency modulates contextual learning in visual search
Source: Psychol Res. 2026 Aug 29;90(5):157. doi: 10.1007/s00426-026-02368-5 (PMC13526094; doi:10.1007/s00426-026-02368-5)
Supplement: Supplementary file 1 [file 426_2026_2368_MOESM1_ESM.docx]

**Sense of Agency and Spatial Learning**

Supplemental materials

## **Data analysis**

***Experiment 1***

We analysed trial-by-trial RTs using linear mixed-effects models (LMMs) implemented in lme4 (Bates et al., 2015). Model specification followed current recommendations for mixed-effects modelling (Barr et al., 2013; Matuschek et al., 2017). We began with a maximal fixed-effects structure containing all main effects and all interactions among log(Epoch), Display, Action Control, and Phase (i.e., all two-, three-, and four-way interactions). The random-effects structure included by-subject random intercepts and random slopes for all within-subject factors. To determine the appropriate fixed-effects structure, we applied a systematic backward-selection procedure using likelihood-ratio tests (LRTs) comparing nested models fit with maximum likelihood (ML). Non-contributing higher-order terms were removed unless their exclusion led to a significant loss of fit.

The LRTs (**Table S1**) indicated that neither the four-way interaction nor any of the three-way interactions improved model fit. However, several two-way interactions were essential: specifically, the terms log(Epoch) × Phase and Display × Control significantly contributed to the model. Because our theoretical focus was on the log(Epoch) × Display × Control interaction (i.e., differences in learning slopes across display types and action control condition), the resulting reduced model thus preserved all lower-order terms structurally necessary for estimating the targeted two-way and three-way interactions while removing unsupported higher-order terms, achieving an appropriate balance between parsimony and explanatory completeness:

*RT ~ 1 + log(Epoch) + Display + Action Control + Phase + log(Epoch): Display + log(Epoch): Phase + Display: Action Control + log(Epoch): Display: Action Control + (1 + log(Epoch)+Action Control + Display + Phase | Participant)*

The results of the reduced model for RT analysis are shown in **Table S2**.

To analyze trial-wise accuracy, we employed a generalized linear mixed-effects model (GLMM) with a binomial link function. Model selection followed a similar procedure to the RT analysis (**Table S3**). To prevent convergence issues, the random-slopes structure was limited to a subset of factors. The final model was specified as follows:

*Accuracy ~ 1+ log(Epoch) + Action Control + Display + Phase + log(Epoch): Display + log(Epoch): Action Control + Action Control: Phase + (1 + log(Epoch)+Display +Phase | Participant).*

Complete results for accuracy analysis are provided in **Table S4.**

**Table S1 Model Comparison (RT) in Experiment 1**

| **Model** | **Description (Removed Term)** | **npar** | **AIC** | **BIC** | **logLik** | **χ²** | **df** | **p** |
| --- | --- | --- | --- | --- | --- | --- | --- | --- |
| Full | log(Epoch) × Display × Control × Phase | 32 | 146132 | 146365 | –73034 | – | – | – |
| No.1 | Removed 4-way interaction | 31 | 146133 | 146359 | –73035 | 2.59 | 1 | 0.107 |
| No.2 | Removed log(Epoch) × Display × Phase | 30 | 146132 | 146351 | –73036 | 1.58 | 1 | 0.208 |
| No.3 | Removed log(Epoch) × Control × Phase | 29 | 146131 | 146343 | –73037 | 1.18 | 1 | 0.277 |
| No.4 | Removed Display × Control × Phase | 28 | 146130 | 146334 | –73037 | 0.59 | 1 | 0.443 |
| No.5 | Removed Display × Phase | 27 | 146128 | 146325 | –73037 | 0.03 | 1 | 0.861 |
| No.6 | Removed Control × Phase | 26 | 146129 | 146319 | –73039 | 3.06 | 1 | 0.08 |
| No.7 | Removed log(Epoch) × Display | 26 | 146129 | 146319 | –73039 | 0 | 0 | – |
| No.8 | Removed log(Epoch) × Control | 26 | 146129 | 146319 | –73039 | 0 | 0 | – |
| No.9 | Removed log(Epoch) × Phase | 25 | 146135 | 146317 | –73042 | 7.94 | 1 | 0.005 |
| No.10 | Removed Display × Control | 25 | 146155 | 146337 | –73052 | 27.46 | 1 | < .001 |

**Table S2 Model Results (RT) in Experiment 1**

|  | **RT** | | |
| --- | --- | --- | --- |
| *Predictors* | *Estimates* | *CI* | *p* |
| (Intercept) | 742.79 | 706.47 – 779.11 | <0.001 |
| log(Epoch) | -25.37 | -60.06 – 9.33 | 0.152 |
| Phase [Training] | 26.43 | 1.94 – 50.91 | 0.034 |
| log(Epoch) × Display [Old] | 2.12 | -14.18 – 18.43 | 0.799 |
| Display [New] ×ControlFree | 15.11 | -2.89 – 33.10 | 0.1 |
| Display [Old] ×ControlFree | -28.51 | -46.32 – -10.71 | 0.002 |
| log(Epoch) × Phase [Training] | -45.23 | -76.70 – -13.77 | 0.005 |
| log(Epoch) × Display [New] × ControlFree | -15.26 | -31.33 – 0.80 | 0.063 |
| log(Epoch) × Display [Old] × ControlFree | -33.98 | -50.01 – -17.95 | <0.001 |
| **Random Effects** | | | |
| σ2 | 42066.78 | | |
| τ00 NSub | 6859.46 | | |
| τ11 NSub.log(Epoch) | 1405.05 | | |
| τ11 NSub.DisplayOld | 1219.56 | | |
| τ11 NSub.ControlFree | 1328.43 | | |
| τ11 NSub.PhaseTraining | 1637.36 | | |
| ρ01 | -0.77 | | |
|  | -0.26 | | |
|  | -0.06 | | |
|  | -0.37 | | |
| ICC | 0.14 | | |
| N NSub | 24 | | |
| Observations | 10816 | | |
| Marginal R2 / Conditional R2 | 0.067 / 0.197 | | |

**Table S3 Model Comparison (Accuray) in Experiment 1**

| **Model** | **Description (Removed Term)** | **npar** | **AIC** | **BIC** | **logLik** | **χ²** | **df** | **p** |
| --- | --- | --- | --- | --- | --- | --- | --- | --- |
| Full | log(Epoch) × Display × Control × Phase | 26 | 3335.7 | 3526.9 | –1641.9 | – | – | – |
| No.1 | Removed 4-way interaction | 25 | 3334 | 3517.8 | –1642.0 | 0.31 | 1 | 0.576 |
| No.2 | Removed log(Epoch) × Display × Phase | 24 | 3332.2 | 3508.7 | –1642.1 | 0.19 | 1 | 0.666 |
| No.3 | Removed log(Epoch) × Control × Phase | 24 | 3332.7 | 3509.2 | –1642.4 | 0.69 | 1 | 0.406 |
| No.4 | Removed Display × Control × Phase | 23 | 3330.9 | 3500 | –1642.5 | 0.21 | 1 | 0.647 |
| No.5 | Removed Display × Phase | 22 | 3329 | 3490.7 | –1642.5 | 0.07 | 1 | 0.793 |
| No.6 | Removed log(Epoch) × Phase | 22 | 3329 | 3490.7 | –1642.5 | 0 | 0 | 1 |
| No.7 | Removed Display × Control | 21 | 3328.4 | 3482.8 | –1643.2 | 1.39 | 1 | 0.238 |
| No.8 | Removed log(Epoch) × Display | 21 | 3328.4 | 3482.8 | –1643.2 | 0 | 0 | < 0.001 |
| No.9 | Removed Control × Phase | 20 | 3337.1 | 3484.1 | –1648.5 | 10.71 | 1 | 0.001 |
| No.10 | Removed log(Epoch) × Control | 21 | 3328.4 | 3482.8 | –1643.2 | 0 | 0 | < 0.001 |

**Table S4 Model Results (Accuracy) in Experiment 1**

|  | **Accuracy** | | |
| --- | --- | --- | --- |
| *Predictors* | *Odds Ratios* | *CI* | *p* |
| (Intercept) | 53.74 | 34.59 – 83.50 | <0.001 |
| log(Epoch) | 1.7 | 1.29 – 2.23 | <0.001 |
| Control [Free] | 1.49 | 0.88 – 2.53 | 0.136 |
| Phase [Training] | 0.21 | 0.13 – 0.36 | <0.001 |
| Display [Old] | 1.19 | 0.90 – 1.56 | 0.219 |
| log(Epoch) × Control [Free] | 0.75 | 0.47 – 1.20 | 0.233 |
| Control [Free] × Phase [Training] | 3.59 | 1.64 – 7.86 | 0.001 |
| log(Epoch) × Display [Old] | 1.01 | 0.76 – 1.34 | 0.954 |
| **Random Effects** | | | |
| σ2 | 3.29 | | |
| τ00 NSub | 0.32 | | |
| τ11 NSub.log(Epoch) | 0.07 | | |
| τ11 NSub.DisplayOld | 0.07 | | |
| τ11 NSub.PhaseTraining | 0.47 | | |
| ρ01 | -0.48 | | |
|  | -0.19 | | |
|  | -0.64 | | |
| ICC | 0.09 | | |
| N NSub | 24 | | |
| Observations | 11520 | | |
| Marginal R2 / Conditional R2 | 0.201 / 0.275 | | |

***Experiment 2***

We also began with a maximal fixed-effects structure containing all main effects and all possible interactions among log(Epoch), Display, Session, and Outcome Presence (in trial n-1) (i.e., all two-, three-, and four-way interactions). The random-effects structure included by-subject random intercepts and random slopes for all within-subject predictors. Following the same backward model-selection procedure as in Experiment 1, we compared nested models using LRTs. As shown in **Table S5**, neither the four-way interaction nor any of the three-way interactions improved model fit. In contrast, two two-way interactions—log(Epoch) × Display and Display × Session—significantly contributed to the model. The final reduced model was therefore:

*RT ~ 1 + log(Epoch) + Display + Session + Outcome Presence* + *log(Epoch): Display + Display: Session + (1 + log(Epoch) + Display + Session + Outcome Presence | Participant).*

Results from the reduced RT model are reported in **Table S6**.

**Table S5	Model Comparison in Experiment 2**

| **Model** | **Description (Removed Term)** | **npar** | **AIC** | **BIC** | **logLik** | **χ²** | **df** | **p** |
| --- | --- | --- | --- | --- | --- | --- | --- | --- |
| Full | log(Epoch) × Display × Session × Outcome Presence (n-1) | 32 | 204172 | 204415 | –102054 | – | – | – |
| No.1 | Removed 4-way interaction | 31 | 204170 | 204406 | –102054 | 0.28 | 1 | 0.598 |
| No.2 | Removed log(Epoch) × Display × Outcome Presence (n-1) | 30 | 204170 | 204398 | –102055 | 1.8 | 1 | 0.18 |
| No.3 | Removed log(Epoch) × Session × Outcome Presence (n-1) | 29 | 204168 | 204389 | –102055 | 0.38 | 1 | 0.538 |
| No.4 | Removed Display × Session × Outcome Presence (n-1) | 28 | 204167 | 204379 | –102055 | 0.27 | 1 | 0.605 |
| No.5 | Removed log(Epoch) × Display × Session | 27 | 204165 | 204371 | –102056 | 0.75 | 1 | 0.386 |
| No.6 | Removed Session × Outcome Presence (n-1) | 26 | 204163 | 204361 | –102056 | 0.05 | 1 | 0.832 |
| No.7 | Removed Display × Outcome Presence (n-1) | 25 | 204161 | 204351 | –102056 | 0.03 | 1 | 0.873 |
| No.8 | Removed log(Epoch) × Session | 24 | 204160 | 204343 | –102056 | 0.8 | 1 | 0.371 |
| No.9 | Removed log(Epoch) × Outcome Presence (n-1) | 23 | 204160 | 204334 | –102057 | 1.44 | 1 | 0.23 |
| No.10 | Removed Display × Session (significant) | 22 | 204174 | 204341 | –102065 | 16.53 | 1 | < .001 |
| No.11 | Removed log(Epoch) × Display (significant) | 22 | 204172 | 204339 | –102064 | 14.01 | 1 | < .001 |

**Table S6	Model Results (RT) in Experiment 2**

|  | **RT** | | |
| --- | --- | --- | --- |
| *Predictors* | *Estimates* | *CI* | *p* |
| (Intercept) | 871.31 | 804.58 – 938.03 | <0.001 |
| log(Epoch) | -37.47 | -51.44 – -23.49 | <0.001 |
| Display [Old] | -37.36 | -58.26 – -16.46 | <0.001 |
| Session [A] | -0.68 | -62.89 – 61.53 | 0.983 |
| Outcome (Present) | -10.21 | -18.99 – -1.43 | 0.023 |
| Display [Old] × Session [A] | -31.74 | -47.03 – -16.45 | <0.001 |
| log(Epoch) × Display [Old] | -22.35 | -34.06 – -10.65 | <0.001 |
| **Random Effects** | | | |
| σ2 | 56016.78 | | |
| τ00 NSub | 34215.44 | | |
| τ11 NSub.log(Epoch) | 993.94 | | |
| τ11 NSub.DisplayOld | 2500.52 | | |
| τ11 NSub.SessionA | 29302.58 | | |
| τ11 NSub.OutcomePresent | 144.71 | | |
| ρ01 | 0.16 | | |
|  | -0.4 | | |
|  | -0.49 | | |
|  | -0.19 | | |
| ICC | 0.35 | | |
| N NSub | 30 | | |
| Observations | 14794 | | |
| Marginal R2 / Conditional R2 | 0.022 / 0.369 | | |
